# Red-Light-Running Crashes’ Classification, Comparison, and Risk Analysis Based on General Estimates System (GES) Crash Database

**DOI:** 10.3390/ijerph15061290

**Published:** 2018-06-19

**Authors:** Yuting Zhang, Xuedong Yan, Xiaomeng Li, Jiawei Wu, Vinayak V. Dixit

**Affiliations:** 1 MOE Key Laboratory for Urban Transportation Complex Systems Theory and Technology, School of Traffic and Transportation, Beijing Jiaotong University, Beijing 100044, China; 15114233@bjtu.edu.cn; 2 Centre for Accident Research and Road Safety-Queensland (CARRS-Q), Institute of Health and Biomedical Innovation (IHBI), Queensland University of Technology (QUT), Kelvin Grove, QLD 4059, Australia; xiaomeng.li@qut.edu.au; 3 Center for Advanced Transportation System Simulation, Department of Civil Environment Construction Engineering, University of Central Florida, Orlando, FL 32801, USA; wjw345178371@knights.ucf.edu; 4 Research Centre for Integrated Transport Innovation (rCITI), School of Civil and Environmental Engineering, University of New South Wales, Randwick, NSW 2052, Australia; v.dixit@unsw.edu.au

**Keywords:** red-light-running crash, crash types, collision scenarios, GES database, classification tree, quasi-induced exposure technique

## Abstract

Red-light running (RLR) has been identified as one of the prominent contributing factors involved in signalized intersection crashes. In order to reduce RLR crashes, primarily, a better understanding of RLR behavior and crashes is needed. In this study, three RLR crash types were extracted from the general estimates system (GES), including go-straight (GS) RLR vehicle colliding with go-straight non-RLR vehicle, go-straight RLR vehicle colliding with left-turn (LT) non-RLR vehicle, and left-turn RLR vehicle colliding with go-straight non-RLR vehicle. Then, crash features within each crash type scenario were compared, and risk analyses of GS RLR and LT RLR were also conducted. The results indicated that for the GS RLR driver, the speed limit displayed the highest effects on the percentages of GS RLR collision scenarios. For the LT RLR driver, the number of lanes displayed the highest effects on the percentages of LT RLR collision scenarios. Additionally, the drivers who were older than 50 years, distracted, and had a limited view were more likely to be involved in LT RLR accidents. Furthermore, the speeding drivers were more likely to be involved in GS RLR accidents. These findings could give a comprehensive understanding of RLR crash features and propensities for each RLR crash type.

## 1. Introduction

A red-light-running (RLR) crash is defined as the event that a driver, inadvertently or deliberately, runs a red light at a signalized intersection and collides with another road user who has the right-of-way [1]. RLR violation has been identified as one of the prominent contributing factors that affect the crashes occurring at signalized intersections. In addition, RLR violation has resulted in substantial numbers of severe injuries and significant property damage [2,3,4,5]. For example, in the United States, at least 697 people were killed in accidents involving red-light running in 2013, while an estimated 127,000 people were injured [6]. According to the National Statistics of Traffic Accident in Thailand 2015, 1702 red-light-running crashes at intersections were reported, or, 1.96% of all police-reported crashes were caused by drivers violating the red light [7,8].

In order to primarily reduce RLR crashes, it is crucial to focus on how to classify the RLR crashes, and why each type of RLR crashes happens. The RLR crash types can be classified based on vehicles’ pre-crash movements. For example, two different directions of straight-through vehicles would lead to right-angle crashes. A straight-through vehicle with an opposing left-turn vehicle may lead to head-on, angle, or opposite sideswipe crashes. A straight-through vehicle and a U-turn vehicle would result in rear-end, angle, right-angle, or same-direction sideswipe crashes [9]. Additionally, different types of RLR crashes may lead to different levels of severity with different expectations. Three different types of RLR behavior are observed in the field. First, the driver intends to run red lights deliberately. Second, the driver takes an inappropriate stop-go decision at the signalized intersection when encountering yellow signal [10,11]. Third, the driver fails to see the traffic light because of distraction or insufficient visibility of the signal.

So far, researchers have done lots of work in analyzing RLR rates and identifying the factors that impacted drivers’ RLR behavior [12,13,14,15,16,17]. In terms of the traffic conditions, larger intersections and higher traffic volumes were associated with higher RLR violation rates [18]. In terms of human behavior, the longer waits are more likely to encourage RLR violation. The shorter cycles provide drivers more opportunities per hour to run a red light at each change [16]. In terms of vehicle types, truck drivers had the highest RLR violation rate (18.2%), followed by small vehicle drivers (12.9%), and then bus drivers (6.9%) [13]. As for the drivers’ characteristics, younger drivers were more likely to run the red light than older drivers, and drivers who drove alone had a higher RLR rate than those who were with other passengers [12]. Moreover, male drivers were more likely to cross the intersection at the onset of red compared to female drivers [17].

Furthermore, research on estimating the likelihood of RLR occurrences with identified influential factors were also conducted [19,20]. For instance, Chen et al. identified RLR rate by fully utilizing the data obtained from stop bar detectors, downstream entrance detectors, and advance detectors [20]. They investigated the relationships between RLR frequency and influential factors, including arriving time at advance detector, approaching speed, headway, gap to the preceding vehicle on adjacent lane, cycle length, geometric characteristics, and snowing. Ren et al. developed a logistic regression model to predict whether drivers would have RLR behavior [5]. Hill used a linear regression model to estimate RLR frequency per hour by using the number of lanes on the subject approach, the number of lanes on the crossing approach, and average daily traffic [19].

To investigate the characteristics of RLR crashes and fully interpret influential factors that are contributing to RLR violations, there are many ways to collect RLR-related data. For example, video cameras are widely used to collect the observational data [21,22]. The advantage of this method is that videos could track and capture drivers’ natural behavior second by second [23]. However, many other factors cannot be collected through the video, such as age, gender, presence of police, work zone, cell phone use, etc. Contrary to the observational methods, the driving simulators could not only extract the drivers’ behavior data, but also examine many other factors [24,25,26,27], such as the effects of yellow light onset time on drivers’ perception response time, and the effects of drivers’ age on perception response time. However, the disadvantage of the driving simulator is that the experiment may be limited in only one specific RLR collision scenario at a time, which could not reflect behavior of RLR violators comprehensively. Recently, the general estimates system (GES) database published by National Highway Traffic Safety Administration (NHTSA), US, becomes one of the most widely used crash databases, which provides comprehensive information of RLR crashes. Specifically, the database can provide the vehicle’s pre-crash movement information, the drivers’ characteristics information (e.g., age, gender, alcohol or drug use), the vehicle type information, the environmental information (e.g., number of lanes, speed limit, speeding, weather, light conditions and visibility obstruction), and the distracted information.

Therefore, the primary objective of this study is to classify RLR crashes based on vehicles’ pre-crash movements by using the GES database. Based on the classifications, the crash features within each type of RLR are compared, and the propensities of different RLR driver behavior are identified. Finally, the results of this study could provide the effective countermeasures for each type of RLR collision to reduce the number of RLR crashes.

## 2. Methodology

### 2.1. Crash Database

The general estimates system (GES) database is published by NHTSA, US. The database obtains its data by the NHTSA collectors in 60 geographic sites across the United States, and by sampling from more than five million police-reported crashes annually [28]. The crash database includes three main parts: accident information, vehicle information, and driver information. The accident information contains crash characteristics and environmental conditions at the time of the crash (e.g., weather and light conditions). The vehicle information describes the vehicles characteristics, such as types of vehicle, number of lanes, speed limit and speeding. The driver information contains drivers’ age, gender, and alcohol or drug use, which describes all persons involved in the crash. By the case number and vehicle number in each database, the information could be linked to each other for each crash so that the RLR crash could be analyzed.

In this study, the RLR crash data were extracted from the GES database between 2002 and 2014. First, the RLR crash data were selected based on the crash reason (running red lights). All the crashes that were related to red-light running were extracted from the GES database. Then, the RLR crashes were restricted to those crashes that only involved two drivers. The reason is that it is better to identify who is responsible for the crash. Finally, 12,707 RLR crashes were extracted from the GES database. The independent variables used in this study are listed in Table 1, including drivers’ age, gender, alcohol or drug use, type of vehicle, number of lanes, speed limit, speeding, weather, light conditions, distracted driving, and visibility obstruction. For simplicity and ease of interpretation of the results, all the variables are classified into categorical variables.

### 2.2. Crash Feature Comparisons for Each RLR Crash Types Based on Classification Tree Method

**Figure 1:**
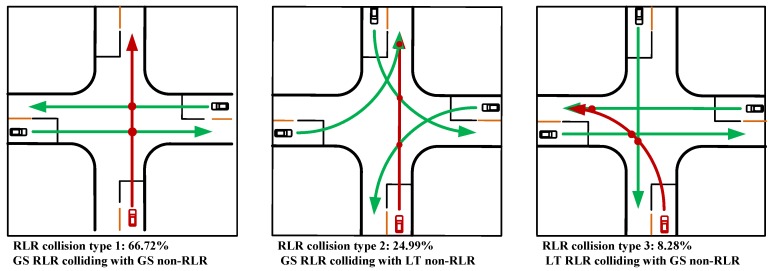
The three red-light-running (RLR) crash types obtained from the general estimates system (GES) database. Note: the green line indicates the approaching path of non-RLR vehicle; the red line indicates the approaching path of RLR vehicle. GS represents vehicle’s go straight movement and LT represents vehicle’s left-turning movement.

In terms of vehicle’ pre-crash movements, statistical results obtained from GES database showed that 88.8% RLR drivers were going straight to cross an intersection, and 8.4% RLR drivers were turning left to cross an intersection. However, the percentage of RLR drivers who were turning right to cross an intersection was lower than others’ pre-crash movements, which was only 2.8%. Also, the right-turn on red is not allowed for all cities in United States, which would lead to the data obtained from GES being difficult to analyze clearly and deeply with regard to right-turn RLR behavior. Thus, only go-straight (GS) and left-turning (LT) drivers were considered in this study. For the GS drivers, drivers who were at fault in an RLR crash were categorized into the GS RLR subgroup; drivers who had no improper driving actions but were involved in non-red-light-running crashes were categorized into the GS non-RLR subgroup. Similarly, in term of drivers’ fault roles, the LT drivers could be divided into LT RLR subgroup and LT non-RLR subgroup. According to the drivers’ pre-crash movements and fault roles, three major typical RLR crash types were summarized, as shown in Figure 1. The RLR crash type 1 represents crashes involving a GS RLR vehicle and a GS non-RLR vehicle, which account for 66.72% of all RLR crashes. The RLR crash type 2 (accounting for 24.99%) represents crashes involving a GS RLR vehicle and a LT non-RLR vehicle, and the RLR crash type 3 (accounting for 8.28%) refers to a LT RLR vehicle and a GS non-RLR vehicle crash. 

**Figure 2:**
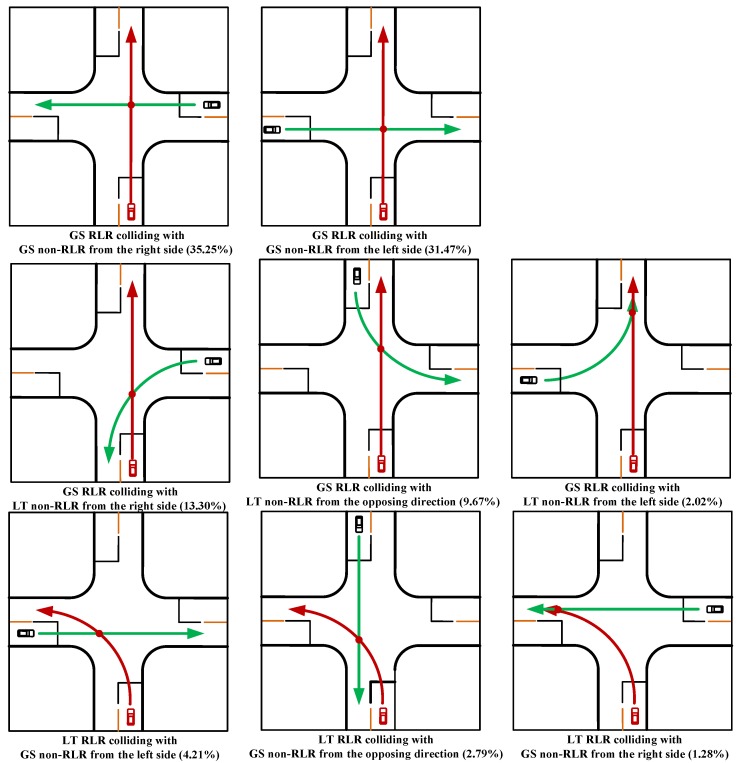
RLR collision scenarios for the three crash types. Note: the green line indicates the approaching path of non-RLR vehicle; the red line indicates the approaching path of RLR vehicle. GS represents vehicle’s go straight movement and LT represents vehicle’s left-turning movement.

Based on the driving direction of non-RLR vehicle, the RLR crash type 1 can be summarized into two scenarios: the GS non-RLR vehicle is approaching from the left side, and the GS non-RLR vehicle is approaching from the right side. Similarly, the RLR crash type 2 includes three scenarios: the LT non-RLR vehicle is approaching from the left side, the LT non-RLR vehicle is approaching from the right side, and the LT non-RLR vehicle is approaching from the opposing direction. The RLR crash type 3 includes three scenarios: the GS non-RLR vehicle is approaching from the left side, the GS non-RLR vehicle is approaching from the right side, and the GS non-RLR vehicle is approaching from the opposing direction. Thus, a total of eight major typical RLR scenarios are summarized based on the three different RLR crash types, which are shown in Figure 2.

In order to compare the crash features for each RLR crash type, three classification tree models were developed. The classification tree model has been proved to be an efficient and explicit interpretable method to explore the relationship between dependent and independent variables, which can straightforwardly yield predictions for the related factors, incorporating the optimal splitting rules in an “if-then” series of statements [29]. In addition, the tree mode is usually presented graphically as hierarchical structures, so that it is easy for people to understand.

### 2.3. Comparison of GS and LT RLR Behavior Propensities Using the Quasi-Induced Exposure Technique

In order to assess the crash risks of GS and LT RLR and compare the GS RLR behavior and LT RLR behavior, the quasi-induced exposure technique was used in this study. The relative crash involvement ratio (RAIR) was used in the quasi-induced exposure analysis as the measure of crash-causing propensity [30,31]. The factors included drivers’ age, gender, and alcohol or drug use, type of vehicle, number of lanes, speed limit and speeding, weather and light conditions, distracted driving, and visibility obstruction. Thus, the RAIR was equal to the ratio of the percentage of a specific subgroup of RLR drivers to the percentage of the same subgroup of non-RLR drivers, and the Equations (1) and (2) for GS and LT group are shown as follows:

(1) $${\mathrm{RAIR}}_{\mathrm{GS},i}=\frac{N1_{GS,i}/\sum N1_{GS,i}}{N2_{GS,i}/\sum N2_{GS,i}},$$

(2) $${\mathrm{RAIR}}_{\mathrm{LT},i}=\frac{N1_{LT,i}/\sum N1_{LT,i}}{N2_{LT,i}/\sum N2_{LT,i}},$$

where ${\mathrm{RAIR}}_{\mathrm{GS},i}$ is the RAIR for drivers of GS group in type *i* condition, $N1_{GS,i}$ is the number of GS RLR drivers in type *i* condition, and $N2_{GS,i}$ is the number of GS non-RLR drivers in type *i* condition. Similarly, ${\mathrm{RAIR}}_{\mathrm{LT},i}$ is the RAIR for drivers of LT group in type *i* condition, $N1_{LT,i}$ is the number of LT RLR drivers in type *i* condition, and $N2_{LT,i}$ is the number of LT non-RLR drivers in type *i* condition.

In addition, the *p*-values generated from a logistic regression qualitatively could be used to show the statistical significance of those RAIR comparisons between different types of drivers, vehicles, or environments [32,33]. Thus, logistic regression model was built to test the significance of RLR crash risk factors based on techniques of induced exposure. In this study, the dependent variable was the RLR crash or not (*Y* = 1 for RLR crashes and *Y* = 0 for non-RLR crashes). The hypothesis testing of logistic regression model was based on the 0.05 significance level. 

## 3. Analysis of RLR Crash Types Based on Classification Tree Models

The classification tree model was used to analyze RLR crash types. The independent variables were used for comparing crash features within each crash type, including type of vehicle, number of lanes, speed limit, speeding, and weather and light conditions. The minimum numbers of cases for parent and child nodes of each classification tree model were both set as 50. The cross-validation method (10-fold) was used to assess the preference value of the tree structure generalizing to a larger population. 

### 3.1. RLR Crash Type #1: GS RLR and GS Non-RLR

**Figure 3:**
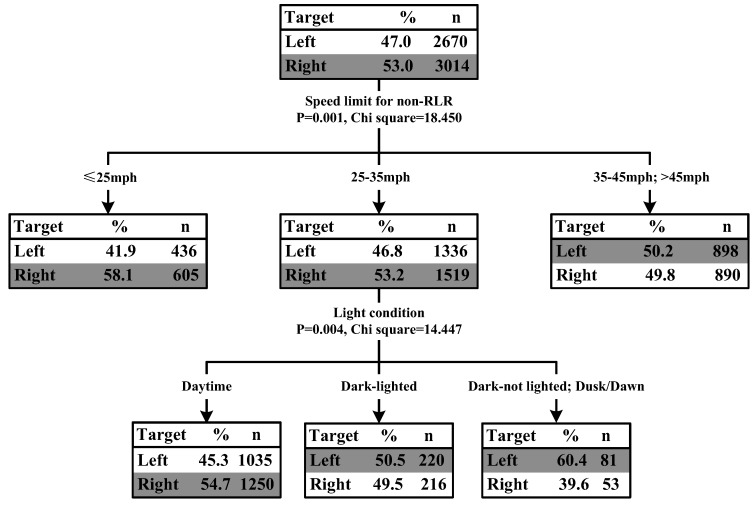
Tree model for RLR crash types #1 go-straight (GS) RLR and GS non-RLR.

The results of classification tree for RLR crash type 1 showed that the speed limit for the road of GS non-RLR vehicle (*p* = 0.001, chi-square = 18.450) and light conditions (*p* = 0.004, chi-square = 14.447) were significantly associated with the percentage of collisions with GS non-RLR vehicle from the right side and left side. Figure 3 illustrated the classification tree diagram for the GS RLR and GS non-RLR crash type.

When an RLR driver was going straight to cross an intersection, the percentage of collisions with GS non-RLR vehicle from the right side was slightly higher than that from the left side (53.0% vs. 47.0%). Generally, if drivers have adequate time to respond to the collision, the possibility of crash occurrence would reduce. For the GS non-RLR drivers in the same situation, the distances to the conflict points between non-RLR vehicle and RLR vehicles from right or left directions seem to be different, and the distance with left side RLR vehicle is smaller than that with right side RLR vehicle (see Figure 1: RLR collision type 1). Thus, the GS non-RLR vehicle would have limited time and space to react to the left side conflicting RLR vehicle, which might be the possible reason of the higher probability of collision between GS RLR vehicle and GS non-RLR vehicle from the right side.

The results also showed that the percentage of collisions with GS non-RLR vehicle from the right side was slightly higher than that from the left side when speed limit for the road of GS non-RLR vehicle was smaller than 25 mph (41.9% vs. 58.1%) or the speed limit was between 25 mph and 35 mph (46.8% vs. 53.2%). When the speed limit for the road of GS non-RLR vehicle was higher than 35 mph, the percentage of collisions with GS non-RLR vehicle from the right side was similar to that from the left side (50.2% vs. 49.8%). One possible reason is that drivers’ reaction time to the conflicting vehicle is insufficient no matter where the RLR vehicle is, due to the higher operating speed.

In addition, when the speed limit for the road of GS non-RLR vehicle was between 25 mph and 35 mph, the light conditions played an important role in crashes with GS RLR and GS non-RLR. Specially, if the RLR crashes occurred during daytime, the percentage of collisions with GS non-RLR vehicle from the right side was higher than that from the left side (45.3% vs. 54.7%); if the RLR crashes occurred at night with light, the percentage of collisions with GS non-RLR vehicle from the right side was similar to that from the left side (50.5% vs. 49.5%). However, if the RLR crashes occurred at night without light, or at dusk/dawn, the percentage of collisions with GS non-RLR vehicle from the left side was higher than that from the right side (60.4% vs. 39.6%).

### 3.2. RLR Crash Type #2: GS RLR and LT Non-RLR

**Figure 4:**
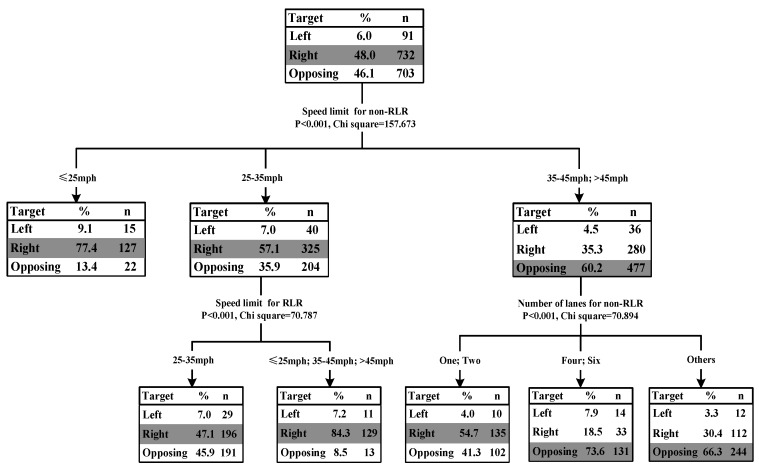
Tree model for RLR crash type #2 GS RLR and left-turn (LT) non-RLR.

The results of classification tree for RLR crash type 2 showed that the speed limit for the road of LT non-RLR vehicle (*p* < 0.001, chi-square = 157.673), speed limit for the road of GS RLR vehicle (*p* < 0.001, chi-square = 70.787) and light conditions (*p* < 0.001, chi-square = 70.894) were significantly associated with the percentage of collisions with LT non-RLR vehicle from the right side, left side, and opposite approach. Figure 4 illustrated the classification tree diagram for the GS RLR and LT non-RLR crash type.

When an RLR driver was going straight to cross a signalized intersection, the percentage of collisions with LT non-RLR vehicle from the right side was highest (48.0%), followed by the collision with LT non-RLR vehicle from the opposing direction (46.1%) and the collision with LT non-RLR vehicle from the left side (6.0%). One possible reason for the highest percentage of collisions with LT non-RLR vehicle from the right side might be related to drivers’ visual field. The right side LT non-RLR driver’s visual field might be obscured by the left hand side A-pillar (windshield frame), which would lead to a blind area from the left of the vehicle path at the entrance to the intersection departure lane [34]. Hence, it would result in the highest percentage of collisions with LT non-RLR vehicle from the right side.

The results also showed that the percentage of collisions with LT non-RLR vehicle from the right side was highest when speed limit for the road of LT non-RLR vehicle was smaller than 35 mph. However, when the speed limit for the road of LT non-RLR vehicle was higher than 35 mph, the percentage of collisions with LT non-RLR vehicle from the opposing direction was highest (60.2%). Compared to the approaching GS RLR vehicle from the left and right side, the relative approaching speed for LT non-RLR vehicle was higher during the approaching process of GS RLR vehicle from opposing direction. Thus, it would be more difficult to avoid the collision with LT non-RLR vehicle from opposing direction due to higher relative speed, and hence would lead to higher percentage of head-on collisions. 

Furthermore, when the speed limit was higher than 35 mph, the number of lanes for the LT non-RLR vehicle showed significant association with the percentages of different collision scenarios for the GS RLR and LT non-RLR crash type. If the number of lanes was equal or less than two, the percentage of collisions with LT non-RLR vehicle from the right side was highest (54.7%); if the number of lanes was higher than two, the percentage of collisions with LT non-RLR vehicle from the opposing direction was highest.

### 3.3. RLR Crash Type #3: LT RLR and GS Non-RLR

**Figure 5:**
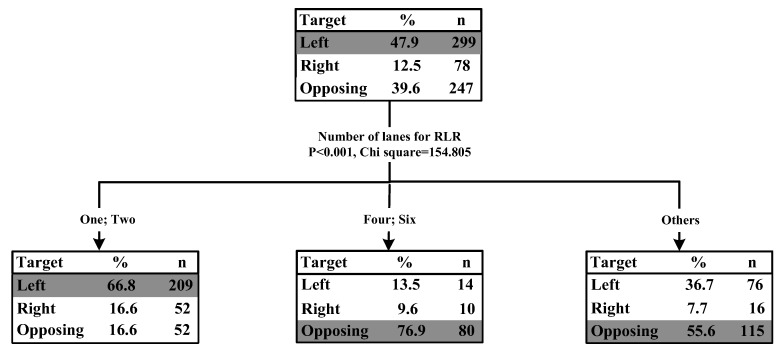
Tree model for RLR crash type #3 LT RLR and GS non-RLR.

The results of classification tree for RLR crash type 3 (LT RLR and GS non-RLR) showed that only the number of lanes for LT RLR vehicle (*p* < 0.001, chi-square = 154.805) was significantly associated with the percentage of collisions with GS non-RLR vehicle from the right side, left side, and opposite approach. Figure 5 illustrated the classification tree diagram for the LT RLR and GS non-RLR crash type. 

The results showed that when an RLR vehicle made a left turn at a signalized intersection, the percentage of collisions with GS non-RLR vehicle from the left side was highest (47.9%), followed by the collision with GS non-RLR vehicle from the opposing approach (39.6%), and the collision with GS non-RLR vehicle from the right side (12.5%). For the LT drivers, drivers’ left visual field might be obscured by the left hand side A-pillar (windshield frame), which would increase the difficulty to detect the left side non-RLR vehicle, and then might lead to a higher percentage of collisions with GS non-RLR vehicle from the left side. 

Additionally, it was found that the number of lanes for LT RLR vehicle played an important role in LT RLR and GS non-RLR crash type. If the number of lanes was equal or less than two, the percentage of collisions with GS non-RLR vehicle from the left side was highest (66.8%); if the number of lanes was higher than two, the percentage of collisions with GS non-RLR vehicle from the opposite direction was highest (76.9% and 55.6%). Previous studies found that the number of lanes influenced the operating speed, and the increased number of lanes would lead to a higher uptrend of the operating speed [35]. As the number of lanes increased, the relative approaching speed between the LT RLR vehicle and GS non-RLR vehicle from the opposing direction also increased, which might lead to higher crash risks. Another possible reason was that more opposing lanes made it more difficult to judge the gap between oncoming vehicles, and hence, increased the collisions with opposing vehicles.

## 4. Results and Discussion of RLR Behavior Propensity Analysis

In this section, quasi-induced exposure technique was used to compare GS and LT RLR behavior propensity, and the results of RLR behavior propensity were shown in Table 2. Furthermore, two logistic regression models were established to test the relationship between factors and relative RLR crash risks obtained from techniques of induced exposure for GS group and LT group, respectively (as shown in Table 3). 

**Figure 6:**
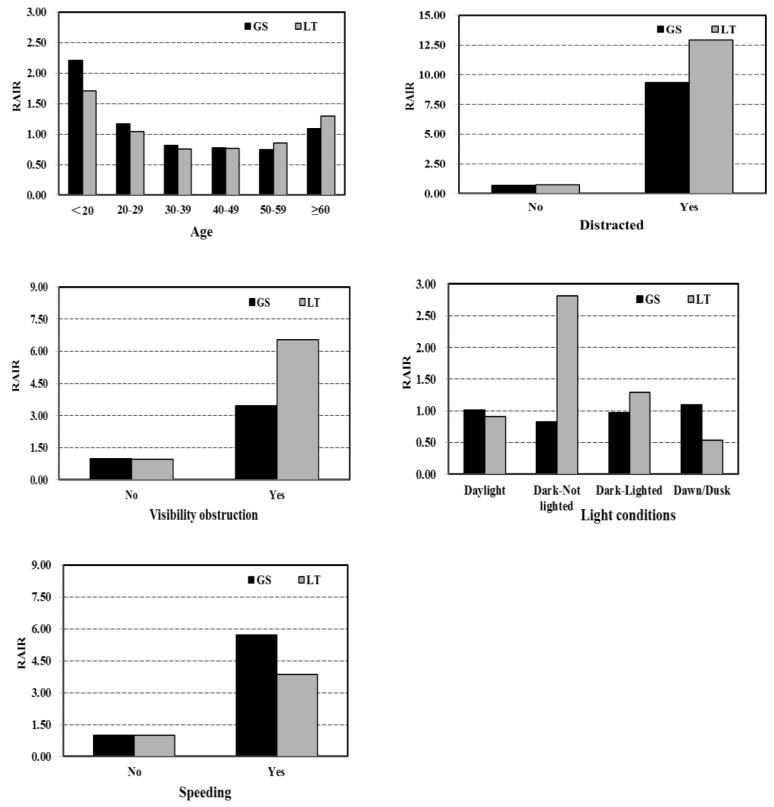
RAIRs for GS and LT groups under different conditions.

For the GS group, drivers’ age (*p* < 0.001), light conditions (*p* = 0.009), distracted driving (*p* < 0.01), visibility obstruction (*p* < 0.001), and speeding (*p* < 0.001) significantly impacted the GS RLR risk. Specifically, the U-shaped pattern of drivers’ age was shown in the GS group (as shown in Figure 6). The young driver group (younger than 20 years old) had the highest RLR risk, followed by the group older than 60 years. The middle-aged groups (30–59 years) had the lowest risk. The possible reason is that young drivers tend to drive more recklessly [36,37]. As for the drivers who are over 60 years old, they might react more slowly to signal changes than younger drivers due to degraded visual ability, deterioration of muscle strength, and reaction time [36]. Therefore, they need more time to make stop–go decisions [38]. For the distracted driving, distractions such as the use of cell phone or other in-vehicle tasks might lead to higher probability of RLR behavior, as distractions could lead to a delayed recognition of critical traffic events while driving [39,40,41]. Also, drivers’ visibility obstruction increased the possibility of RLR behavior, as well as the poor light conditions. If the drivers’ visual field is restricted or visibility is low, they may not have timely perception of the yellow or red signal, and then break the proper space cushion that provides drivers enough reaction time to slow down before the stop line at the onset of red signal [42,43]. For the speeding drivers, they were more likely to run red lights which might be due to the longer distance required to stop. Besides, factors such as number of lanes (*p* < 0.001), speed limit (*p* < 0.001) had been found to influence the possibility of GS RLR significantly as well. For the GS drivers, the increased number of lanes would increase the possibility of RLR violations, as well as the speed limit. 

Drivers’ age (*p* = 0.024), light conditions (*p* < 0.001), distracted driving (*p* < 0.001), visibility obstruction (*p* < 0.001), and speeding (*p* = 0.011) were significant factors for the LT RLR group, which was also shown in Table 2 and Table 3. The possible reason is similar to the GS group. In addition, it was found that vehicle types (*p* = 0.023) had significant effects on the LT RLR possibility. For the LT RLR crashes, heavy vehicle drivers were more likely to run red lights. Due to a complex vehicle structure and operation requirement, heavy vehicle drivers normally need longer stop distances and more space to perform a turn, which might motivate the driver to pass through the intersection rather than stop. In addition, the longer time and increased effort required by the heavy vehicles to accelerate after stopping for a red light might also lead to the heavy vehicle driver’s RLR behavior [44,45].

Furthermore, there were significant differences between drivers’ age, distracted driving, visibility obstruction, light conditions, and speeding in the GS and LT RLR risks. As shown in Figure 6, drivers who were over 50 years old, distracted drivers, drivers who had obstructed visibility, or drivers who were under dark conditions were more likely to have an LT RLR crash. In addition, the speeding drivers were more likely to be involved in GS RLR crashes. Compared to the GS drivers, the LT drivers usually decelerate to change to the exclusive left turn lane, or the shared left-turn/through lane in advance, and observe the signal and the surrounding traffic at the same time, which might need more effort in risk detection and response. In consideration of the complex operations involved in left-turn, the older drivers would require a longer time to detect and react to signal changes [21,36]. Adverse driving conditions, such as distraction, limited field of view, and poor light conditions would exacerbate the difficulty in performing left-turn. Eventually, the weak detecting ability and limited visual conditions lead to an increase in LT RLR crash risk. For the speeding drivers, they tended to underestimate the time and distance that the vehicle required to stop from a high speed in a straight-forward approach situation. Consequently, speeding drivers were more likely to involve in GS RLR crashes.

## 5. Conclusions

This study presented three RLR crash types with consideration of vehicles’ pre-crash movements (GS RLR vehicle colliding with GS non-RLR vehicle, GS RLR vehicle colliding with LT non-RLR vehicle, and LT RLR vehicle colliding with GS non-RLR vehicle) based on the crash data obtained from the GES database. For each RLR crash type, two or three RLR collision scenarios were categorized according to different approaches of the non-RLR vehicle. In order to compare crash features for different RLR collision scenarios within each crash type and identify the effects of factors related to vehicle and environment on the likelihood of crash occurrence, three corresponding classification tree models were developed. The results showed that (1) for the GS drivers, the probability of collision with non-RLR vehicles from the right side was highest; (2) when the speed limit for non-RLR vehicle was larger than 35 mph, the probability of collision with GS non-RLR vehicles from the right side was highest, and the probability of collision with LT non-RLR vehicles from the opposing side was highest; (3) for the LT driver, the probability of collision with GS non-RLR vehicles from the left side was highest; (4) when the number of lanes for LT RLR vehicle was no less than two, the probability of collision with GS non-RLR from the opposing direction was highest. Moreover, the results of risk analyses of GS and LT behavior indicated that the drivers who were older than 50 years, or distracted, or had a limited view, were more likely to be involved in LT RLR crashes, and speeding drivers were more likely to be involved in GS RLR crashes. According to the results of this study, appropriate engineering countermeasures need to be considered to reduce RLR crashes. For the intersection design and operation, a sufficient sight distance could help drivers reduce reaction times and detect traffic signal or potential conflicting vehicles in time. For the light conditions, the illumination contrast of signal lights and surrounding environment should be taken into account to enhance the visibility of signal lights and reduce the crash occurrence rate. Furthermore, warning information could also be used to provide advance information to drivers about signals ahead, especially in adverse weather conditions.

However, the limitations of this study should be noted. As the GES data come from a range of different police investigations, the reliability problem of the database has been raised by many traffic experts. However, the GES database has been widely used in many studies as an empirical traffic crash database for exploring crash characteristics, and it was often demonstrated as being reliable [46]. Nevertheless, it is still suggested that the same study be conducted using a different empirical database, and that the results be compared. Secondly, for some influencing factors, such as visibility obstruction and distraction, the data obtained from GES database could not provide complete samples for each specific classification of these factors. It is recommended that future research conduct these studies using driving simulators to explore the systematic influence mechanism of different visibility obstruction and distraction. Thirdly, because of the limited information of traffic phase, the effects of traffic signal related factors (such as phase and length) on the LT RLR behaviors are not discussed. Finally, more human factors could be considered in future research, such as drivers’ reaction time, which might be very important in analyzing drivers’ RLR behaviors propensity.

## Figures and Tables

**Table 1:** Independent variables used in this study.

Variables	Definition	Categorical Values
Age	Indicates drivers’ age at the time of the crash. Six groups are divided in this study.	<20
		20–29
		30–39
		40–49
		50–59
		≥60
Gender	Indicates the sex of drivers involved in the crash.	Male
		Female
Alcohol or drug use	Indicates whether alcohol/drug use involved in crash.	No
		Yes
Types of vehicle	Indicates a classification of vehicle based on its general configuration.	Car
		Light vehicle
		Heavy vehicle
		Others
Number of lanes	Indicates the number of travel lanes.	One
		Two
		Four
		Six
		Others
Speed limit	Indicates posted speed limit in miles per hour. Four groups are divided in this study.	<25 mph
		25–35 mph
		36–45 mph
		>45 mph
Speeding	Indicates whether the driver’s speed is larger than the posted speed limit.	No
		Yes
Weather	Indicates the atmospheric conditions. Two groups are divided in this study.	Clear
		Adverse
Light conditions	Indicates the type/level of light that exists at the time of the crash.	Daylight
		Dark—Not lighted
		Dark—Lighted
		Dawn/Dusk
Distracted	Indicates whether the driver is distracted before the crash occurs.	No
		Yes
Visibility obstruction	Indicates whether the driver’s vision is obstructed before the crash occurs.	No
		Yes

**Table 2:** Relative crash involvement ratio (RAIR) comparisons between GS group and LT group.

Factors	GS Group			LT Group		
	NON-RLR%	RLR%	RAIR	NON-RLR%	RLR%	RAIR
Age						
<20	6.26%	13.82%	2.209	8.57%	14.61%	1.705
20–29	21.52%	25.07%	1.165	20.86%	21.77%	1.044
30–39	19.79%	16.15%	0.816	18.90%	14.21%	0.752
40–49	19.89%	15.35%	0.772	20.00%	15.31%	0.765
50–59	16.66%	12.33%	0.740	15.66%	13.42%	0.857
≥60	15.89%	17.27%	1.086	16.01%	20.68%	1.292
Distracted						
No	96.54%	67.68%	0.701	97.77%	71.18%	0.728
Yes	3.46%	32.32%	9.337	2.23%	28.82%	12.931
Visibility obstruction						
No	98.80%	95.86%	0.970	98.89%	92.72%	0.938
Yes	1.20%	4.14%	3.457	1.11%	7.28%	6.547
Light conditions						
Daylight	78.23%	78.94%	1.009	78.64%	71.85%	0.914
Dark—Not lighted	3.29%	2.72%	0.827	2.20%	6.20%	2.813
Dark—Lighted	15.51%	15.08%	0.972	15.46%	19.98%	1.292
Dawn/Dusk	2.97%	3.27%	1.098	3.70%	1.97%	0.533
Speeding						
No	99.71%	98.33%	0.986	99.80%	99.22%	0.994
Yes	0.29%	1.67%	5.696	0.20%	0.78%	3.841

Note: RLR represents red-light running. RAIR represents drivers’ relative crash involvement ratio. GS represents vehicle’s go straight movement and LT represents vehicle’s left-turning movement.

**Table 3:** Logistic regression results of RLR crashes for GS group and LT group.

Factors	df	GS Groups			LT Groups		
		B	Sig.	Exp (B)	B	Sig.	Exp (B)
Age (<20)	5	---	0.000	---	---	0.024	---
Age (20–29)	1	−0.648	0.000	0.523	−0.396	0.087	0.673
Age (30–39)	1	−0.959	0.000	0.383	−0.721	0.006	0.486
Age (40–49)	1	−1.073	0.000	0.342	−0.728	0.004	0.483
Age (50–59)	1	−1.084	0.000	0.338	−0.537	0.038	0.584
Age (≥60)	1	−0.646	0.000	0.524	−0.227	0.361	0.797
Distracted (Yes)	1	2.850	0.000	17.292	3.016	0.000	20.406
Visibility obstruction (Yes)	1	1.650	0.000	5.206	1.357	0.000	3.884
Light conditions (Daylight)	3	---	0.009	---	---	0.000	---
Light conditions (Dark—Not lighted)	1	−0.261	0.069	0.770	1.254	0.000	3.505
Light conditions (Dark—Lighted)	1	−0.198	0.004	0.821	0.528	0.002	1.695
Light conditions (Dawn/Dusk)	1	0.098	0.474	1.103	−0.342	0.382	0.711
Speeding (Yes)	1	1.945	0.000	6.990	1.981	0.011	7.252
Number of lanes (One)	4	---	0.000	---	---	---	---
Number of lanes (Two)	1	0.627	0.010	1.872	---	---	---
Number of lanes (Four)	1	0.985	0.000	2.679	---	---	---
Number of lanes (Six)	1	1.320	0.000	3.742	---	---	---
Number of lanes (Others)	1	0.819	0.001	2.268	---	---	---
Speed limit (≤25 mph)	3	---	0.000	---		---	
Speed limit (25–35 mph)	1	0.164	0.034	1.178	---	---	---
Speed limit (35–45 mph)	1	0.387	0.000	1.473	---	---	---
Speed limit (>45 mph)	1	0.201	0.058	1.223	---	---	---
Vehicle types (Car)	3	---	---	---	---	0.023	---
Vehicle types (Light vehicle)	1	---	---	---	−0.192	0.281	0.826
Vehicle types (Heavy vehicle)	1	---	---	---	0.599	0.012	1.820
Vehicle types (Others)	1	---	---	---	−0.117	0.528	0.889
Constants	1	−0.452	0.081	0.637	−1.788	0.000	0.167

Note: df represents degrees of freedom. B represents coefficient value. Sig. represents *p* value. Exp (B) represents odd ratio.
